# Lie for Me: Developmental Trends in Acquiescing to a Blatantly False Statement

**DOI:** 10.3389/fpsyg.2021.691276

**Published:** 2021-09-23

**Authors:** Amelia Courtney Hritz, Stephen J. Ceci

**Affiliations:** Department of Psychology, Cornell University, Ithaca, NY, United States

**Keywords:** interview, false memory, witness, age, compliance, lie

## Abstract

A pair of studies demonstrates that simply asking children to make a blatantly false accusation in the guise of helping others can result in both immediate and long-term false claims. In the pilot study, the initial willingness to make a blatantly false statement was associated with some children making false statements a week later despite being told that the first interviewer had made mistakes during the initial interview. On a positive note, the majority of participants accurately stated that they did not have first-hand knowledge of their accusation's accuracy. Across both studies, the rate of false accusation rates was high. The main experiment demonstrated that children who were young, possessed the lowest verbal intelligence or who were from the lowest SES homes made the most accusations. These findings illustrate not only the dangers of encouraging children to make false statements, but the ease and durability of making such false statements.

## Introduction

A large corpus of research has documented the deleterious effects of various interviewing behaviors. This research has demonstrated that children's report accuracy can be reduced as a result of providing either pre- or post-event misinformation. Pre-event misinformation that can damage children's report accuracy includes the provision of false stereotypes, rumors, inaccurate co-witness information, and unfulfilled expectations (e.g., Pynoos and Nader, 1989; Leichtman and Ceci, 1995; Garven et al., 2000; Principe et al., 2006). Similarly, post-event misinformation that can damage children's report accuracy includes misleading suggestions provided by an interviewer (e.g., Poole and Lindsay, 1995; Cassel et al., 1996), visualization inductions (Ceci et al., 1994), reinforcement (Garven et al., 1998, 2000), imagination-inflation techniques, and leading questions (Ceci, 1994; Ceci and Bruck, 1998; Bruck et al., 2006; Howe et al., 2009; Otgaar et al., 2016, 2017).

In addition to these suggestive techniques associated with report inaccuracy, a growing body of research has documented developmental trends in children's willingness to mislead interviewers. Talwar, Lee, and their associates have produced a large body of research on the developmental course and cognitive and social correlates of lie-telling (e.g., Talwar and Lee, 2002a,b, 2008; Talwar et al., 2007a,b; Talwar and Crossman, 2011; Evans and Lee, 2013). Lies told in the service of achieving selfish ends, such as gaining material rewards or escaping punishment, begin early during the pre-school years and are reduced in magnitude by middle childhood, whereas lies told in the service of socially desirable ends, such as pretending to appreciate an undesirable gift, tend to begin later. Both types of lies are associated with cognitive and social factors such as theory of mind, social skills, and parenting (e.g., Clarke-Stewart et al., 2004; Lavoie et al., 2016).

The current study builds on the developmental research on children's report inaccuracy and deception in several ways: first, we examine a context that differs from spontaneous lies generated to serve selfish or prosocial aims, namely, willingness to acquiesce to a blatantly false persuasion request, using a paradigm that has not been used for this purpose previously but which may have practical relevance to legal cases. Second, we are interested in the intersection between lies and false beliefs, asking whether the former can influence the latter as some have opined (e.g., Leichtman and Ceci, 1995; Zaragoza et al., 2001; Otgaar and Baker, 2018). Finally, we include sociodemographic variables that have only rarely been included in the memory development literature, but which theoretical research suggests could be important sources of systematic developmental variability (Bronfenbrenner and Ceci, 1994; Ceci et al., 2010). Very few studies have examined children's acquiescence to a misleading persuasion as a function of parent social class, which Talwar, Lee and their colleagues suggest may moderate children's acquiescence. We also include the role of verbal intelligence as a potential moderator; although it has received some attention (e.g., Roebers and Schneider, 2001; Chae and Ceci, 2005), it has heretofore not been studied in the context of acquiescence to blatantly false persuasion requests. The closest it has come to this context was a study by Clarke-Stewart et al. (2004) that found that 5-year-olds with the highest verbal intelligence were the most resistant to adult attempts at false persuasion.

Children may make statements about events they initially know to be false if the statements are suggested by adults who hold *a priori* beliefs about their authenticity. Adults may signal their beliefs through methods such as repeating specific misinformation during questioning (e.g., Warren et al., 1991; Leichtman and Ceci, 1995; Poole and Lindsay, 2001; Moore et al., 2018), offering praise, bribes or threats of punishment (Garven et al., 1998; Schreiber et al., 2006), rejecting or ignoring children's denials (White et al., 1997; Garven et al., 1998), and selectively reinforcing their incorrect statements (Zaragoza et al., 2001). Such social pressure can cause a child to make statements that, while consistent with the belief of the interviewer, are inconsistent with the child's actual perceptual experience (for a review see Ceci and Bruck, 1998; Bruck et al., 2006). Even mild forms of suggestion can increase inaccurate reports by children, such as descriptions of events by parents (e.g., Thompson et al., 1997; Poole and Lindsay, 2001), visualization inductions (Ceci et al., 1994) being informed that co-witnesses have made a disclosure (Principe and Ceci, 2002), stereotypes (Leichtman and Ceci, 1995; Moore et al., 2018), or even naturally-occurring conversations with parents and peers (Bruck et al., 1999; Principe and Schindewolf, 2012; Kim et al., 2017).

Once encoded, false memories can lead children to maintain inaccurate reports in later neutral interviews. Efforts to retrieve accurate memories following the creation of false memories—for instance by instructing children to say when they are unsure, or correcting the interviewer when she makes a false suggestion—often will not offset the impact of the false memory (e.g., Poole and Lindsay, 2001; Zaragoza et al., 2001), although there is some evidence that warning children that questions may be tricky does result in a small but significant reduction in errors (~5%) to suggestive questions (Warren et al., 1991). Even efforts to talk children out of their false beliefs can be unsuccessful (e.g., Ceci et al., 1994; Ceci, 1995; Leichtman and Ceci, 1995; Kim et al., 2017). And the metacognitive strategies that are useful in rejecting false information—recollection rejection, retrieval editing, and monitoring—are less effective for young children (Brainerd et al., 2008; Moore et al., 2018). These past findings set the stage for the current study by moving the issue from subtle misleading suggestions by an interviewer to blatantly false persuasive statements.

In contrast to the voluminous literature on the effects of suggestive interviews and misinformation [e.g., see integrative reviews by Poole and Lamb (1998); Quas et al. (2000); Otgaar et al. (2014); Schneider (2015); Goodman et al. (2017)] as well as on factors associated with developmental trends in lie-telling (e.g., the work of Talwar, Crossman, Lee and their colleagues), we were interested in what would happen if instead of exposing participants to techniques designed to bias their report accuracy—such as suggestive interviews, stereotypes, visual inductions, post-event misinformation, incentives to lie—an adult blatantly makes a false accusation in the guise of helping others. Hence, in the current research, we were interested in (a) what happens when children are exposed to an adult's blatantly false assertion and they are asked to repeat it and sign their agreement with it, an extreme form of forced confabulation. Related questions include: (b) will children acquiesce and sign an adult's blatantly erroneous statement, and if so, (c) will they subsequently incorporate it into their long-term reports to a neutral interviewer who instructs them to ignore the blatantly incorrect prior interviewer, and (d) what, if any, developmental trends or social or cognitive correlates will influence their performance?

Thus, in the following experiments we sought to determine whether will children make a blatantly false accusation merely because someone asks them to do so to help unnamed others? And, if so, will children later maintain the false accusation when interviewed outside the presence of the previously biased adult by an unbiased person who urges them to report only what they actually experienced, not what the blatantly biased adult told them? We hypothesized that (a) children will be more likely to make a false accusation in an initial interview if they are blatantly asked to do so in service of helping unnamed others (children who attended a different school, they were told), and (b) they will subsequently maintain this false assertion with a neutral interviewer, especially if they are younger and more suggestible and/or have lower verbal intelligence. These were all *a priori* expectations that we made based on previous research showing that the mechanisms that could drive this effect are unfolding rapidly over early and middle childhood as we briefly describe below. We undertook a mini test of the planned procedure to make sure it would work with the intended age groups in a larger-scale experiment with controls; this mini test gave us confidence that the youngest children understood the procedure (e.g., the wording to request them to endorse a blatantly false statement) although it also led to changes in the procedure.

There are empirical and theoretical reasons for positing that the period between early and middle childhood is one of rapid development of the factors of interest in the current study. A confluence of cognitive, social, and neurobiological developments unfold between early and middle childhood that are relevant to understanding the effect of exposure to blatantly false statements on later report accuracy. Specifically, social developments are occurring between the ages of 4 and 10 that could be relevant in the context of children's compliance with false persuasions by an adult. As noted, younger children are more influenced by a powerful adult authority figure than are older children (e.g., Ceci et al., 1987); preschoolers are more inclined to conform to false persuasion than are adults (Clarke-Stewart et al., 2004) but older children are actually less likely to conform than adults, thus a U-shaped function (Kim et al., 2017). There are also improvements in source monitoring over early and middle childhood (e.g., Poole and Lindsay, 1995). Quas et al. (2000) reported that younger children had significantly more difficulty sourcing their memories than did older children, a skill that is relevant in the present study in which children are asked to describe an event to a neutral interviewer after receiving a false persuasion request from a prior interviewer, thus creating a potential source misattribution. On the other hand, in their extensive review, Bruck and Melnyk (2004) did not find correlations between source monitoring and suggestibility-proneness even though both displayed normative developmental trends (Quas et al., 2000); the majority of the studies Bruck and Melnyk reviewed showed no relationship between individual differences in source monitoring and suggestibility. Young children also lack metamemorial insights that limit their recollective accuracy, as shown in Wellman's (1978) classic work, when they were more impeded by misinformation (for review see Ceci and Bruck, 1993). Preschool-aged children also lack strategy knowledge, such as elaborative rehearsal (Ornstein, 1978), and they have relatively undeveloped retrieval-time editing (Brainerd and Reyna, 2001), and second-order theory of mind in which participants must identify a third person's beliefs based on what the second person believes, which does not asymptote before at least age 8 (e.g., Arslan et al., 2012; Hiatt and Trafton, 2015). Although the right dorsolateral region of the pre-frontal cortex continues to develop into young adulthood, it is disproportionately undeveloped among preschoolers (Giedd et al., 1999; Gogtay et al., 2004), limiting their ability to inhibit, track, and monitor the contents of their memory (Ceci et al., 2010). In short, this confluence of developments in cognitive (memory, strategy use, theory of mind), social (conformity, deferral of memory to those of perceived as more authoritative), and neuromaturational (tracking, monitoring) mechanisms converge to anticipate that the period between early and middle childhood is one of heightened relevance for the present hypotheses. It was for this reason that we focused on this transition.

The departure point for the present set of experiments is that unlike studies that employed techniques known to bias report accuracy, in the present study none of the documented explicit or implicit suggestive techniques that damage report accuracy were employed—i.e., there were no post-event suggestions, misleading questions, visually-guided inductions, stereotypes, reinforcements, or automatic semantic associations that have previously been shown to cause children's reporting errors (Bruck and Ceci, 1999; Ceci and Friedman, 2000; Ceci and Bruck, 2006). Instead, the present approach seems straightforward and could have implications for disclosures that come about in the context of one adult urging a child to acquiesce to a false statement about another. For example, in an acrimonious custody dispute a child, upon the request of one parent, may initially repeat a blatantly false statement made by one parent about another parent, and over time elaborate the statement, as has been suggested by some to occur in actual cases (see Bruck and Ceci, 2013). What effect might repeating a blatantly false assertion have on a children's later reports to a neutral interviewer who encourages report accuracy?

## Pilot Study

Based on insights from a demonstration study of 16 children who watched clowns perform magic tricks, we designed a somewhat larger pilot study in which children watched a chemist perform “magic tricks” with chemistry. An interviewer later asked children to tell them that the chemist had broken a test tube during the demonstration. At no time did the chemist break any of the test tubes in the magic show and children were not shown any evidence of a broken test tube during either the event itself or during either of the two interviews that followed it. Thus, the children in this experiment were not provided with any evidence that the chemist broke a test tube during the show they watched; the only basis for claiming he did so was an assertion by the initial interviewer that others who attended a different camp session had told her he broke a test tube at their session.

### Methods

#### Participants

Data for this pilot study were collected on 54 children attending a summer camp hosted by a children's science museum. These participants ranged in age from 6 to 11 years old (*M* = 8.52, *SD* = 1.42); 41% were male. Ideally, we planned to extend this age range downward to capture the entire early childhood-middle childhood span that has been implicated in the developmental research reviewed earlier.

#### Procedure

The measures and procedure employed in this study were approved by the university's institutional review board (1303003677 “Children's False Accusations without Suggestive Questioning”). Participants watched a chemist perform 12 different magic tricks including a trick where she mixed acids, bases, and indicators in a test tube to create a rainbow tube. Several days later (*M* = 2.02 days, *SD* = 0.94), participants were interviewed individually by an unfamiliar female adult. This interviewer solicited a free narrative about what transpired during the magic show, followed by two directive questions about the magic show: “Do you remember seeing a test tube?”; “Did the chemist do any tricks with the test tube?” (Answers to these questions and the child's free narrative served as a baseline to judge later responses that followed this interviewer's request to endorse her blatant lie). Next, the interviewer informed the child that she had heard from children who attended another chemistry show that the chemist broke his test tube during their show. Then she blatantly asked the child to say the chemist broke a test tube during the magic show they saw (even though he had not). Specifically, she said: “I need your help so he doesn't accidently do this again, or other children will not get to see the magic show. Can you help me? Can you tell me the chemist broke the test tube so I can make sure he doesn't do this again?” The direct request for affirmation was done to resemble a situation in which an adult asks a child to affirm a non-event, one they could not have witnessed but which they might assume the adult had witnessed and needed their assent for prosocial reasons (allow future children to see the show). After the child answered, the interviewer thanked the child and offered stickers.

Approximately 1 week later (*M* = 7.56 days, *SD* = 6.38), a neutral interview took place during which an unfamiliar female adult explained that the previous interviewer had made mistakes when talking to children about the magic show, and she wanted the child to report only what they actually saw in the magic show rather than what the previous interviewer told them. Following these instructions from this neutral interviewer, she asked the child if the chemist accidentally broke his test tube. If the child said yes, the interviewer asked if they had seen it happen with their own eyes. Following this neutral interview, children completed intelligence and suggestibility measures.

In light of the literature on verbal ability and suggestibility, we administered the vocabulary subset of the Wechsler Abbreviated Scale of Intelligence (Wechsler, 1999) to examine if a g-loaded measure of children's verbal intelligence predicted responses during the first and second interview. The vocabulary scaled scores from the Wechsler series of intelligence tests were collected as a rough measure of general intelligence, given its very high saturation on the general intelligence factor, g (Flynn, 2007; Nisbett, 2009). Raw scores on the vocabulary subset of the Wechsler Abbreviated Scale of Intelligence were calculated based on the general scoring principles (Wechsler, 1999). These raw scores were converted to T-scores, which are age-corrected (thus removing any correlation with chronological age) and have a wide range (Wechsler, 1999). Results indicated that intelligence was normally distributed (*M* = 63.53, *SD* = 11.44, *n* = 53) with negative skew.

We administered the Video Suggestibility Scale for Children (VSSC) to discover whether suggestibility predicted responses during the first and second interview. The VSSC produces two parameters of suggestibility-proneness: yield (succumbing to erroneous statements) and shift (changing originally correct answers to false answers in response to negative feedback) (Scullin and Ceci, 2001; Scullin et al., 2002). Results did not differ when the “yield” and “shift” measures from the VSSC were examined separately, and therefore the combined parameter scores were used for the analysis. Scores on the VSSC were normally distributed with a slight positive skew (*M* = 9.6, *SD* = 4.35, *n* = 52).

We found no correlation among our individual difference measures: verbal intelligence and suggestibility were not correlated, and suggestibility was also not correlated with age.

### Results

Most participants were willing to accuse the chemist of breaking the test tube, even though they had not witnessed it and were not presented with any physical evidence of a broken test tube. When blatantly asked to make a false statement, 34 out of 54 children (63%), asserted that the chemist broke the test tube. Importantly, no child made such false allegations spontaneously during their prior free narrative; it was only done in response to the subsequent blatant request from the interviewer. One week later after being told the initial interviewer made mistakes and got children to make mistakes about what happened during the magic show, most children recanted. Only 13 of the 34 children who had previously asserted the chemist broke the test tube maintained their false accusation. All but two of the children who asserted the accusation during the second interview had also made the assertion in the first interview. These children who made false accusations in both interviews were 32% of the 34 people who made the accusation in the first interview and 20% of the total original sample of 54. In addition, five children asserted that they saw the chemist break the test tube with their own eyes. This represented 16% of the 34 who made the accusation and 9% of the original 54.

In sum, a fraction of the participants who watched the chemist do a magic show went along with the initial interviewer's blatant request to affirm her false allegation and many subsequently maintained this false allegation with some claiming to have witnessed it with their own eyes despite no suggestive techniques being employed during the second interview (i.e., there was no provision of erroneous post-event information, misleading questions, imagery inductions, requests to speculate, clumsy stereotypes, forced confabulation questions, etc.). In contrast to the children who went along with the first interviewer's blatant request for a false affirmation, none of the 54 children had made such false allegations spontaneously in their free narratives of what occurred before the interviewer requested the child make a false affirmation. Thus, the damage to children's report accuracy was the result of children assenting to the initial interviewer's assertion, a small fraction of whom subsequently claimed when speaking with a neutral interviewer a week later not only that it occurred but to have witnessed it with their own eyes.

We next compared the children who made a false accusation by age, verbal intelligence, and suggestibility-proneness. Results are displayed in Table 1. As we expected, the youngest children appeared to make most of the false allegations. Because it is possible that with more participants, age would emerge as a significant predictor, we tested this in the main experiment, using a predetermined sample size that possessed ample power to detect differences of the observed magnitude. Similarly, as noted, ultimately a total of five children in this Pilot Study who watched the chemistry show said they saw with their own eyes the chemist break the test tube, despite being told by the neutral interviewer that the initial interviewer had caused children to make mistakes and that they should only report what they actually witnessed rather than what the prior interviewer may have told them. All five of these children were 8 years old and younger. Even with the small sample size in this pilot, this age difference was reliable, *t*(51) = 2.02, *p* < 0.05, Hedges' *g* = 0.95 (large effect). Further comparisons of these five participants did not lead to additional significant results, which could be due to the small sample size.

**Table 1:** Pilot study: average age, intelligence, and suggestibility by whether they made an accusation during the first interview, second interview, or both.

	**Two accusations**	**T 1 accusation only**	**No accusation**
Age	7.81 (1.09)	8.76 (1.59)	8.66 (1.13)
Intelligence	62.20 (6.84)	64.05 (11.80)	63.6 (14.1)^a^
Suggestibility	9.82 (5.17)	10.00 (4.21)^a^	9.06 (4.30)
*N*	11	22	18

*Values displayed are means (standard deviations in parentheses). Two participants made an accusation in the second interview but not the first, due to the small number they are excluded from the table*.

a *1–2 participants missing scale data*.

We did not notice trends when we compared children who made a false accusation by verbal intelligence or suggestibility. Follow-up work with larger and more diverse (in terms of verbal intelligence) samples was conducted in the following main experiment to provide a more robust test of these findings.

## Main Experiment

The data and the findings from pilot study revealed a number of interesting results despite the small sample size. In view of these results, the main experiment was a modification designed to broaden the context by substituting a more active role for the child than was the case in the pilot study where the child passively observed a magic show, and also to include another dependent variable: parent socio-economic status (Talwar et al., 2017). In this experiment children were once again asked by an interviewer to agree with a blatantly false assertion but in a less passive context from the one used in the pilot study. Because of the narrow age differences in the pilot study, this experiment was designed with finer age gradations to shed light on developmental vs. reverse developmental effects (e.g., Kim et al., 2017). Once again, vocabulary scaled scores from the Wechsler series of intelligence tests were collected as a rough measure of general intelligence, g (Flynn, 2007; Nisbett, 2009). Finally, we endeavored to recruit a broad range of SES given that compliance with adult authority figures may be related to parent educational attainment as well as suggestibility (Chae et al., 2016).

### Method

#### Participants

Children were recruited through schools, preschools, and a university-run summer camp. One hundred and seventy-one children and adolescents participated, 43 4-year-olds (23 females; *M* = 50.40 months, *SD* = 2.53), and 44 6-year-olds (25 females; *M* = 73.70 months, *SD* = 3.86), 44 8-year-olds (22 females; *M* = 99.09 months, *SD* = 5.02), and 44 12-year-olds (19 females; *M* = 151.27 months, *SD* = 6.27).

#### Procedure

This experiment involved one male and one female research assistant. Children were brought into a testing room and greeted by an opposite-sex research assistant. The youngest children were escorted by a parent or guardian and the older children were escorted by a teacher's aide or a camp counselor. The opposite-sex assistant provided crayons and a coloring book for the child to play with for ~10 min. Toys were displayed prominently on the table, as well as several items of clothing, including a straw hat. After ~10 min, a same-sex research assistant entered the room and was introduced to the child as Jenn or John by the opposite-sex assistant who then departed. To avoid cross-sex confounds, all final interviews were conducted by same-sexed research assistants. After entering the room, the same-sex assistant engaged the child in actively playing a couple rounds of Simon Says. During this game Jenn or John instructed the child “Simon Says [John/Jenn] put on a straw hat,” which the same-sex assistant donned; they went through five such actions. After playing this game, the original opposite-sex assistant returned to the room and the same-sex assistant departed. After several minutes of amiable interaction with the child, the opposite-sex research assistant asked the child two memory questions related to real and suggested actions in the Simon Says game (“Do you remember the name of the person who played Simon Says with you?,” “Do you remember what Simon Says told him (or her) to do with the straw hat?”). Next, this opposite-sex assistant made two blatantly false assertions about events: the child was told that John (or Jenn) had broken a non-present cell phone and asked the child to say that they saw this happen. Following their response, they were presented with a typewritten document and asked to make a mark to indicate if they saw John (or Jenn) break the cell phone. Then they were told that John (or Jenn) ripped a colored drawing and they were asked to make a mark to indicate that they observed John (or Jenn) rip it. In reality, John (or Jenn) did neither thing (Older participants were asked to sign their name on a line indicating they saw John (or Jenn) rip the drawing and break the cell phone; pre-school-aged children were shown two blank spaces on the sheet where they were asked to make crayon marks to indicate that they saw John (or Jenn) damage each of these items, if they agreed that this happened).

#### Individual Differences

To measure verbal intelligence, raw scores on the vocabulary subtest of the Wechsler Preschool and Primary Scale of Intelligence (for the youngest age group) and the Wechsler Intelligence scale for Children-IV (for the three older groups) were converted to scaled scores with a mean of 10 and a standard deviation of 3 based on national norms by age. As was true in the pilot study, the sample was skewed slightly above the national average, with a mean of 11.11, and standard deviation of 2.52.

Parent educational attainment is reported in Table 2. Parent SES may moderate the effect of parenting practices on children's willingness to lie (Talwar et al., 2017) and has been tied to report accuracy and suggestibility (e.g., Chae et al., 2016). After coding parent education on a six-point scale and averaging across both parents, parent education was strongly correlated with children's verbal intelligence scaled scores, *r*(173) = 0.37, *p* < 0.001.

**Table 2:** Parent education of participants.

	**Parent 1**	**Parent 2**
Some high school or less	1	0
High school diploma/GED	22	10
Some college	35	23
College degree	46	51
Some graduate school	48	58
Graduate degree	22	28
Unreported	1	5

Participants were asked two questions about salient details in the Simon Says game to gauge how well they attended to it. The questions probed the name of the assistant with whom they played Simon Says (Jenn/John) and what was placed on their partner's head (a straw hat). Based on the accuracy of their answers, they were assigned values of 0, 1, or 2. Thirteen participants answered both questions incorrectly (7%), 68 answered one correctly (39%), and 94 answered both correctly (54%).

For each age group descriptive information on the individual difference variables is displayed in Table 3.

**Table 3:** Descriptive statistics by age group.

	**4 year olds**	**6 year olds**	**8 year olds**	**12 year olds**
Intelligence	10.98 (3.01)	11.18 (2.58)	11.23 (2.22)	11.05 (2.26)
Parent Education	4.19 (1.33)	4.32 (1.08)	4.09 (1.04)	4.19 (0.96)
Accuracy				
One correct	22 (51%)	16 (36%)	12 (27%)	18 (41%)
Two correct	17 (40%)	25 (57%)	29 (66%)	23 (52%)
*N*	43	44	44	44

*For intelligence, parent education and accuracy, values are mean (standard deviation), all other values are sample size. Parent education is coded on a six-point scale and averaged across parents: 1, did not complete high school; 2, high school graduate; 3, some college attendance; 4, college completion; 5, some post-baccalaureate coursework; 6, PhD or professional degree*.

### Results

One-hundred-forty-five participants claimed to have witnessed at least one false event, and 66 participants claimed to have witnessed both false events. A logistic regression model was estimated to predict which children made a false assertion as a function of age, verbal intelligence, and parent education. We mean-centered age, standardized intelligence, and mean-centered the average parent education. Table 4 shows the results of the regression. As can be seen, age and parent education were significant predictors for making at least one false assertion. Younger children and children whose parents had less education were more likely to make at least one false assertion. We estimated a 41% increase in the odds of making a false accusation for a 1-year decrease in age.

**Table 4:** Logistic regression predicting signing at least one false statement.

	***B***	***SE***	**95% CI**	**Odds**	**Odds 95% CI**
Intercept	−2.59^***^	0.40	−3.48, 1.89	0.75	0.03, 0.15
Age	0.34^***^	0.08	0.19, 0.51	1.41	1.21, 1.67
Intelligence	0.22	0.29	−0.33, 0.80	1.25	0.72, 2.23
Parent's education	1.59^***^	0.37	0.92, 2.39	4.89	2.51, 10.90

*** *p < 0.001*.

*Post-hoc* analysis further confirmed the strong relationship between age and making false assertions. When we substituted the continuous age variable with the age group variable in the logistic regression, keeping the other predictor variables the same, and ran pairwise comparisons, the 12-year-olds were significantly less likely to make a false assertion compared to all other age groups. More specifically, compared to 12-year-olds, the odds of making a false assertion were 14.35 times higher for 4-year-olds, 12.29 times higher for 6-year-olds, and 5.13 times higher for 8-year-olds (*p*'s = 0.002, 0.003, 0.05, respectively). Thus, like the pilot study, this study also documented age differences.

There was a similarly strong relationship between parent education and false assertions. As displayed in Table 4, we estimated the odds of signing a false statement were 4.89 times higher for each unit decrease in parent education. In fact, all of the children who refrained from signing either of the false statements had parents with at least a baccalaureate degree. Of the children whose mothers did not have a college degree, 66% signed both false statements (compared to 33% in the group whose mothers had a college degree and 17% in the group with mothers with post baccalaureate education). A similar trend was found with father's education.

Intelligence was not a significant predictor, however, as noted, it was significantly correlated with parent education. When parent education was omitted from the regression, intelligence was significant, *p* = 0.05.

We noticed that there may be a relationship between accuracy and making a false assertion: of the 13 participants who answered both salient questions incorrectly, all claimed to have witnessed at least one false event and 8 of 13 claimed to have witnessed both (62%). In case the 13 participants who answered both questions about salient details incorrectly were not sufficiently paying attention, we reran the regression after omitting their responses and results were not significantly different: age and parent education remained significant predictors of making false assertions (both *p*s < 0.001).

In addition, we ran a logistic regression comparing individuals who made both false assertions to individuals who made one or zero false assertions. Again, in this regression age and parent education were significant predictors (both *p*s < 0.001).

## Discussion

In the pilot study a sizable portion of children complied with an adult's request to make a blatantly false accusation even though they lacked first-hand knowledge of the alleged infraction. On the other hand, most children who made these false accusations did accurately disclose the truth in a subsequent neutral interview after the interviewer gave them releasing instructions (“The person who talked to you before made a lot of mistakes and got children to make mistakes …. Please tell me only what you actually saw, not what someone told you.”). And even when they complied with the blatant request to make a false statement and maintained this falsehood when subsequently interviewed by a neutral interviewer, most of these children did not claim to have seen it with their own eyes. In sum, these findings demonstrate that under conditions in which the child is only subjected to a single blatant request for false information, in later interviews a small number of the young children may purport to remember the accusation and misattribute its source to personal experience observing the infraction rather than to the blatantly false statement by the initial interviewer who asked them to claim they actually observed it.

After the neutral interviewer informed children that the prior interviewer had made mistakes and misled children, not all children accurately disclosed that they had not observed the infraction. It is possible that children's initial compliance may have created false memories, retrieval competitions, or source misattributions in some of the youngest children who claimed to have seen the infraction with their own eyes despite being given “release” instructions by the neutral interviewer. Such release instructions should, if anything, have motivated them to retract their former false assertion if they were aware of its falsity. Of course, this is speculative as we have no direct test of the hypothesis that the initial compliance request actually distorted memory as opposed to other possibilities such as children's loyalty to, or even fear of, the adult in the first session, could plausibly lead to them to continuing lying in the follow-up interview. Future research will be needed to test this.

In contrast to reversed developmental trends in which younger children are more resistant to spontaneous false memory due to their less developed semantic associative networks (e.g., Brainerd et al., 2010; Brainerd and Reyna, 2012; Otgaar et al., 2016; Kim et al., 2017), in both the pilot and main study, false assertions were more likely among the youngest children. This developmental finding is consistent with well-documented age trends in source misattributions which routinely document that younger children have greater difficulty separating various sources or inputs into their memories (e.g., Ceci et al., 1994; Quas et al., 2000; Goodman et al., 2017). This literature suggests that children who make source misattributions genuinely come to believe in the veracity of their misattributions and “generally were not able to report that they had been asked about these events in prior interviews” (Quas et al., 2000, p. 218).

In the main study, false assertions were found to occur disproportionately more often among children who had the least educated parents. Relatively little research has examined children's acquiescence to misleading suggestions as a function of socioeconomic and intellectual factors. In the present study, a proxy for parent social class proved to exert a powerful influence. In light of this, one wonders whether prior findings in the developmental literature might be qualified if future researchers were to replicate former designs but include a socioeconomic measure. The role of verbal intelligence has received some attention (e.g., Roebers and Schneider, 2001; Chae and Ceci, 2005). This literature suggests that children with higher verbal intelligence provide more accurate recall and children with very low intelligence can be more suggestible in response to misleading questions. In our study, intelligence, which was significantly correlated with parent education, did not uniquely predict making a false assertion in the context of conformity to blatant lies.

Taken together, the findings from these experiments have implications for cases in which adults articulate biases during conversations with children. The influence of the interviewer can lead some children to make accusations that they initially know are false. In itself, this is hardly a new finding, as decades of deception research have documented that children will lie in response to various incentives (e.g., Talwar et al., 2011; Wyman et al., 2016). However, the present findings demonstrate that this is more strongly observed among the youngest participants from the lowest educated families.

### Caveats and Limitations

The results of the pilot study and main experiment are limited in their forensic implications because we refrained from creating the stress associated with an actual forensic interview in which children: (a) usually know the individual they are accusing, (b) understand that their answers may influence others' opinion of this individual, and (c) are cognizant that this individual could face adverse consequences as a result of their statement. Ethical considerations preclude us from making children feel seriously uncomfortable or protective of loved ones. Thus, the children in these experiments were told that it was probably an accident that the item was broken, and that their help was needed to make sure this did not happen again so that children in another school could enjoy the use of the item. This was done to minimize stress but at the same time it deviates from legal contexts where stress is inherent, thus limiting its practical import.

Furthermore, there were no negative consequences associated with making a false accusation. There was also far less pressure on the children to make false accusations than may inhere in child abuse cases in which multiple suggestive methods might be used in interviews, such as introduction of new suggestive information, positive reinforcement, interviewer's expression of disbelief when a child fails to disclose, conformity pressure, and invitations to pretend or speculate (e.g., Garven et al., 1998; Schreiber et al., 2006; Bruck and Ceci, 2013). If in real cases interviewers with biases exerted more pressure and used more suggestions than was done in the current study, the deleterious consequences could be even greater than what were observed under these less intensive circumstances. Thus, even though a substantial fraction of the children in these experiments affirmed the false statement and maintained this affirmation over time, this might have been elevated by increasing the intensity and number of requests.

In addition, the present experiments, although careful to include variables that have often been missing from past studies (socioeconomic status, verbal intelligence), nevertheless did not examine other potentially important individual differences that could be instrumental. Recently, a number of researchers have begun to examine such factors as child and parental attachment status as it relates to suggestibility (Chae et al., 2014, 2018), children's frontal neurological status (Poole et al., 2014), children's social skills (Lavoie et al., 2016), and parental rearing styles as they relate to children's compliance with a false report (Kim et al., 2017). It would be interesting for future work to add such variables to the study of blatantly false statements.

Future research will have to chart the boundary conditions of this effect, although some experimental evidence indicates that children's suggestibility is exhibited even under conditions of stressful physical experience such as during painful medical procedures (e.g., Bruck et al., 1995), and case studies of contested custody are rife with analogs of the present procedure. For example, Bruck and Ceci (2013) describe a custody case that progressed into a series of accusations of sexual abuse by the father of two preschool-aged daughters. The children likely overheard claims made by their mother and repeated statements to a counselor such as “Mommy says Daddy is mean.” The present findings suggest that initially agreeing with an adult's request to affirm such assertions may result in some children later repeating it to a neutral interviewer even if the child initially was aware they had not witnessed them (e.g., in the above case the allegation that the father had harshly snatched a credit card from the mother and cut it half).

## Data Availability Statement

The raw data supporting the conclusions of this article will be made available by the authors, without undue reservation.

## Ethics Statement

The studies involving human participants were reviewed and approved by IRB, Cornell University. Written informed consent to participate in this study was provided by the participants' legal guardian/next of kin.

## Author Contributions

AH and SC designed the study together. SC drafted the introduction and discussion. AH drafted the methods and results. The manuscript has been read and approved by both authors. Once the full manuscript was assembled, both authors contributed to editing and finalizing the manuscript. Both authors contributed to the article and approved the submitted version.

## Conflict of Interest

After the writing of this article but before publication, AH became employed by the law firm Shapiro Arato Bach LLP. The remaining author declares that the research was conducted in the absence of any commercial or financial relationships that could be construed as a potential conflict of interest.

## Publisher's Note

All claims expressed in this article are solely those of the authors and do not necessarily represent those of their affiliated organizations, or those of the publisher, the editors and the reviewers. Any product that may be evaluated in this article, or claim that may be made by its manufacturer, is not guaranteed or endorsed by the publisher.
